# Case Report: SARS-CoV-2 Infection—Are We Redeemed? A Report of Candida Spondylodiscitis as a Late Complication

**DOI:** 10.3389/fmed.2021.751101

**Published:** 2021-11-26

**Authors:** Luis Miguel Moreno-Gómez, Olga Esteban-Sinovas, Daniel García-Pérez, Guillermo García-Posadas, Juan Delgado-Fernández, Igor Paredes

**Affiliations:** Department of Neurosurgery in University Hospital “12 de Octubre”, Madrid, Spain

**Keywords:** COVID-19, candida, spondylodiscitis, corpectomy, antifungal therapy

## Abstract

**Background:** We describe a case of candida spondylodiscitis secondary to coronavirus disease (COVID-19).

**Case report:** A 47-year-old man diagnosed with severe acute respiratory syndrome coronavirus 2 (SARS-CoV-2) required prolonged admission to the intensive care unit (ICU). Four months later, he was diagnosed with thoracic candida spondylodiscitis. Medical management was insufficient, so he eventually underwent surgery.

**Discussions:** Fungal infections seem to be more likely in patients with COVID-19, but it is unknown whether they are directly attributed to COVID-19 or other surrounding factors. Regardless of the answer, the diagnosis is complicated, and the mortality rate is high.

**Lessons:** COVID-19 is posing a challenge to the society, and new and unexpected diseases that had once disappeared have risen again. It is our duty to suspect them and to treat them in the most effective way possible.

## Introduction/Background

Spinal infections are rare, representing around 1% of all bone infections and comprising <2 per 10,000 of all hospitalizations in tertiary care centers (1, 2). Factors associated with this disease include diabetes, renal disease on dialysis, cirrhosis, chronic immunosuppression, AIDS, and intravenous drug users (2). Most of these infections have a pyogenic or tuberculosis origin (1, 3), and infections of fungal cause are scarce. They are associated with immunosuppressive states and prolonged stays in intensive care units (ICUs), among other conditions (1, 2, 4–6). Despite their low prevalence, they should not be neglected given their specific management and non-negligible mortality (7, 8).

Currently, there are few reports about fungal spondylodiscitis (9, 10). Thus, we present a case report of this unfamiliar infection with the particularity of affecting a patient with a prolonged ICU stay due to infection with severe acute respiratory syndrome coronavirus 2 (SARS-CoV-2). We describe his medical condition thoroughly and review the literature about the after-effects of COVID-19, fungal spondylodiscitis, and its treatment.

## Case Report

A Hispanic 47-year-old man was admitted to our emergency department in March 2020 with a 2-day-long clinical condition of fever, cough, and dyspnea. He was diagnosed with coronavirus disease (COVID-19) acute respiratory infection and was admitted to our hospital (Table 1). His medical history described him as hypertensive, obese, and occasional drinker. He did not report other medical diseases, including serology for hepatotropic viruses, HIV, and other parasitic infections negative. He had no relevant surgical, familiar, psychosocial, or genetic history.

**Table 1:**
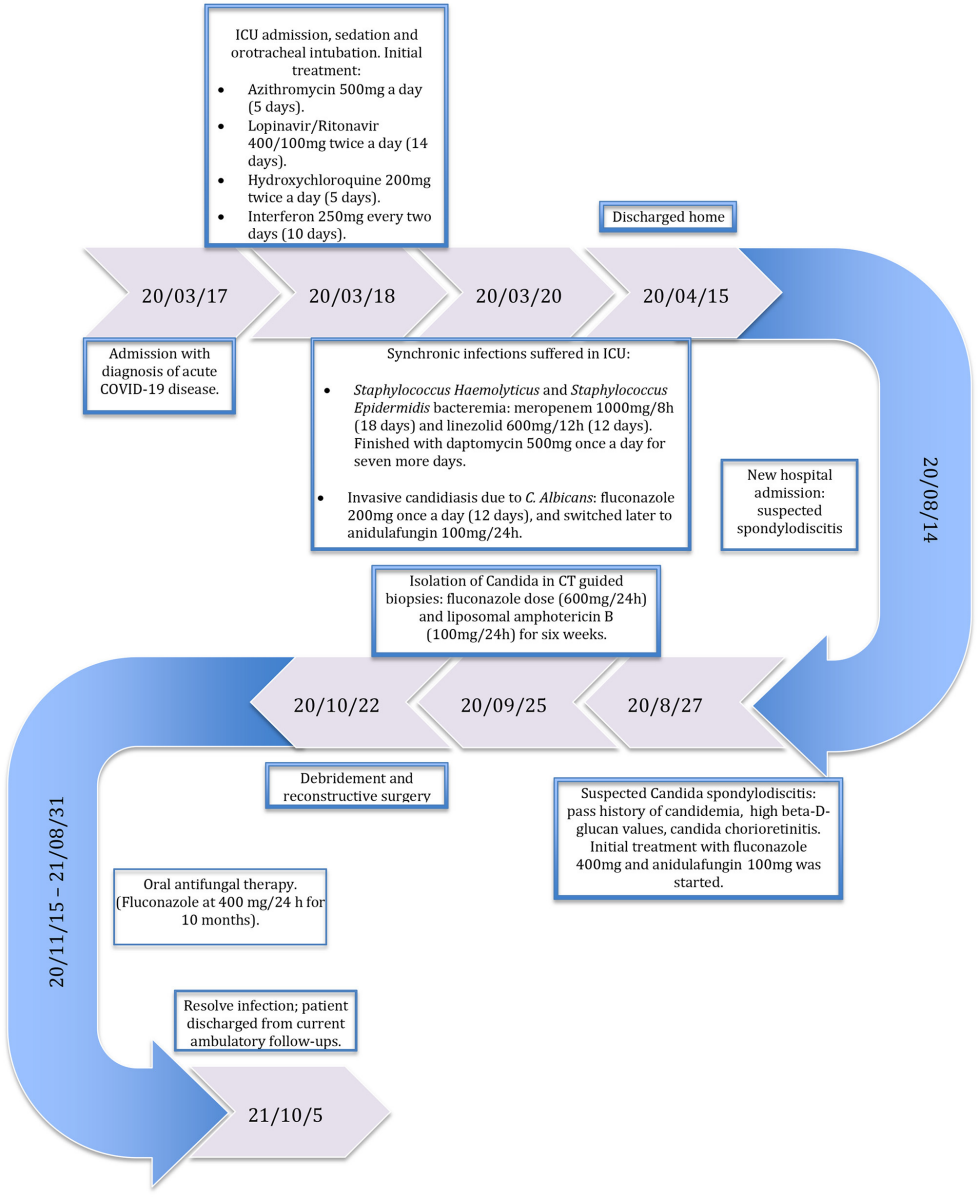
Subject's timeline.

In the following days, he experienced worsening of his respiratory disease and required ICU admission with sedation and orotracheal intubation. The initial treatment started with the following:

- Azithromycin: 500 mg per day for 5 days.
- Lopinavir/ritonavir: 400/100 mg every 12 h for 14 days.
- Hydroxychloroquine: 200 mg every 12 h for 5 days.
- Interferon: 250 mg every 2 days for 10 days.

Despite this treatment, the patient suffered the following complications:

- *Staphylococcus haemolyticus* and *Staphylococcus epidermidis* bacteremia, which was treated with meropenem at 1,000 mg every 8 h for 18 days and linezolid at 600 mg every 12 h for 12 days. The treatment was finished with daptomycin at 500 mg once a day for seven more days.
- Invasive candidiasis due to *Candida albicans*, which was isolated in multiple blood, central venous system device, and bronchoalveolar samples and was related to prolonged use of intravascular devices. It was initially treated with fluconazole at 200 mg once a day for 12 days and later switched to anidulafungin at 100 mg daily.
- Myopathy and neuropathy due to long-lasting critical care.

The patient was discharged 1 month later, by which the problems had resolved, and negative results were obtained in microbiological analysis. Four months later, the patient was referred for intense dorsal back pain without fever. A routine follow-up thoracic CT was performed, and the results were suggestive of D8-D9 spondylodiscitis. No neurological impairment was identified, but his initial level of C-reactive protein (CRP) (2.88 mg/dl) and beta-D-glucan values were high, so he was admitted again to our hospital for further tests.

**Figure 1:**
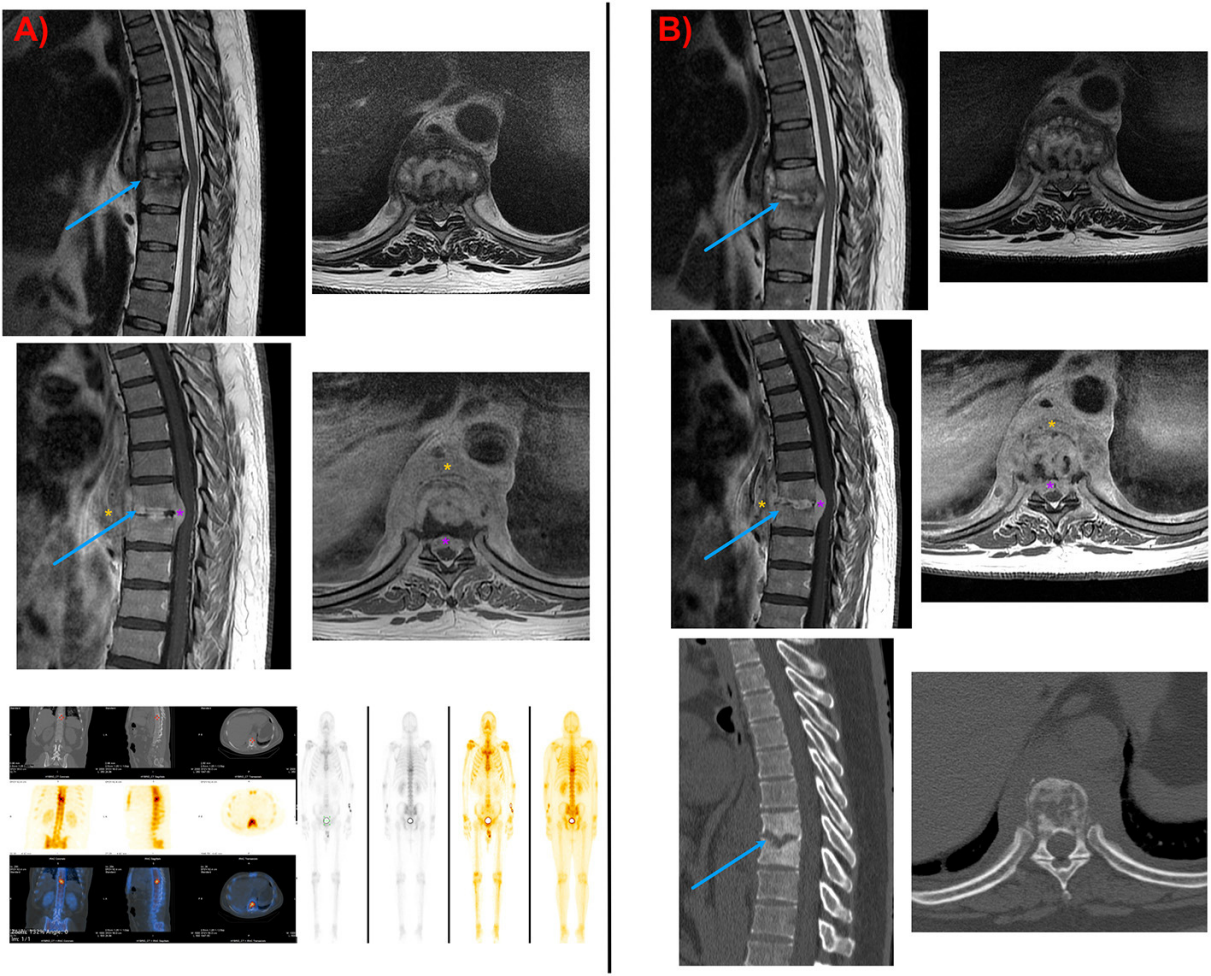
**(A)** Initial MRI image. Note the important prevertebral mass with disc and adjacent bone involvement typical of fungal spondylodiscitis. In addition, an epidural mass was seen with spinal cord compression. Positive SPECT-TC and gamma scan showing a pathological uptake where spondylodiscitis was suspected. **(B)** MRI and CT were taken a few days before surgery. The results reaffirmed the necessity of surgery in this case, given the inefficiency of medical treatment. Note the worsening of the Kyphosis curve, the damaged bone, and the epidural mass. Blue arrow, spondylodiscitis destruction; Orange asterisk, prevertebral mass infection; Purple asterisk, epidural mass infection.

Serial MRIs showed destruction of intervertebral space D8-D9 and the disc. A single-photon emission computerized tomography (SPECT) and gamma scan confirmed the highly suggestive diagnosis of spondylodiscitis (Figure 1A). Despite initial cultures and microbiological samples being negative, due to his history of candidemia, antifungal treatment was started with 400 mg of fluconazole and 100 mg of anidulafungin. No clinical response was noticed, and the inflammatory markers and beta-D-glucan values continued to rise, so vertebral samples were taken. Ultimately, *C. albicans* was isolated, for which we started a new treatment with a higher dose of fluconazole (600 mg/24 h) and liposomal amphotericin B (100 mg/24 h) for 6 weeks. Surgery was performed due to a lack of improvement in serial MRIs and blood inflammatory markers (Figure 1B).

**Figure 2:**
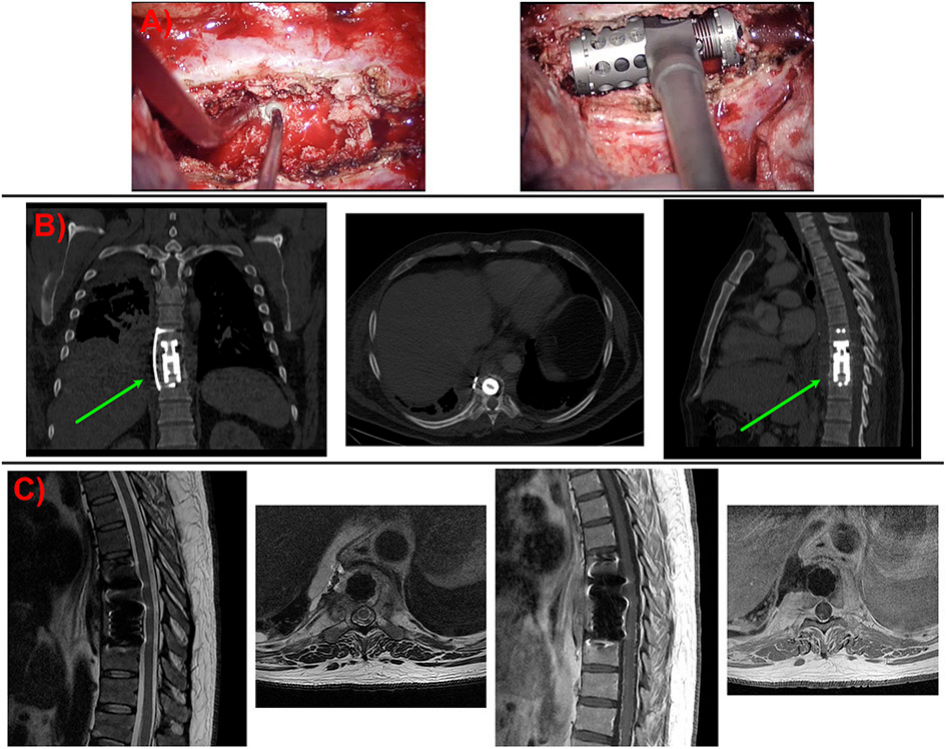
**(A)** Intraoperative images. Pathological bone in the affected vertebrae was drilled with a high-speed drill. Once the vertebrectomy was done, an expandable cage was fitted inside. **(B)** Coronal, axial, and sagittal CT views after surgery. No complications related to the approach or osteosynthesis materials were identified. **(C)** MRI images were taken after surgery. Note the evacuation of the epidural mass and the decompression done on the spinal cord in the axial views. Green arrow, osteosynthesis material (expandable titanium cage with a lateral fixation plate).

The patient was placed under general anesthesia with selective lung intubation using a double-lumen tube. He was placed in a left lateral decubitus position, and a right lateral transthoracic approach was performed through the T7 intercostal space. Once the thoracotomy was done, the right lung was collapsed, and the vertebrae were reached *via* a transpleural approach (Figure 2A). X-ray was used to confirm the correct positioning of the hardware, and then the right lung ventilation was resumed. A chest tube drainage was left in place, and the wound was closed.

The postoperative course was uneventful. The dorsal pain improved markedly, and the patient was discharged home with a Jewett brace and oral antifungal therapy (fluconazole at 400 mg/24 h for 10 months). During follow-up, the patient completed full-dose antifungal therapy. After 8 months of follow-up, the infection seemed to have resolved, the patient had fully recovered, and he returned to his normal life (Figures 2B,C).

## Discussions

The quick spread of the new SARS-CoV-2 infection has become a global health emergency. Compared with other common respiratory viruses, such as influenza, this disease has had higher numbers of complications requiring ICU admission (11). To the best of our knowledge, the case presented is the first report of candida spondylodiscitis secondary to SARS-CoV-2 infection that required surgical treatment.

Candida-induced spondylodiscitis is an extremely rare infectious complication of the spine and represents <5% of the spondylodiscitis cases reported (12). The frequency of candida infection has risen lately due to the increasing number of patients who are at risk, immunocompromised, and under high antibiotic pressure (mostly from suffering a fungal infection). In this case, the use of intravascular devices, the treatment with broad-spectrum antibiotics and immunomodulatory drugs, and the compromised immunological function associated with COVID-19 infection (13) played an important role in the initial candidemia (11).

It has been suggested that the rate of invasive fungal infections is higher in patients with SARS-CoV-2 intensive care (14, 15), and these patients are most likely to develop pulmonary aspergillosis, oral or hematogenous candidiasis, or pneumocystis pneumonia. Nevertheless, it is still unknown whether bacterial and fungal infections are directly attributable to SARS-CoV-2 (16). Other factors should also be considered, such as the overwhelming number of critically ill patients, prolonged duration of mechanical ventilation/critical care admission, and an overstretched health-care system. Our patient was in the ICU for 24 days, and he had received treatment with hydroxychloroquine, lopinavir/ritonavir, interferon, azithromycin, and ceftriaxone during this period. Furthermore, due to high levels of inflammatory markers and fever, he received meropenem and linezolid for 3 weeks.

The prevalence of COVID-19-associated co-infections varies between series. However, the prevalence of secondary infections could be as high as 50% among non-survivors. The difficulty of early diagnosis of co-infections during COVID-19 pneumonia has become a challenge (11, 17, 18). It is also important to take into account that the mortality related to invasive nosocomial candida infection can be up to 40% (19, 20). There is a lack of guidelines about antifungal prophylaxis and treatment in patients with severe COVID-19 pneumonia who require mechanical ventilation (16).

A delay in treatment is common because of the difficulty in diagnosis, which can lead to serious sequelae (21), and there are no clinical or radiological characteristics specific to this infection (8). However, Simeone described four predictors in the differential diagnosis of fungal and *Staphylococcus aureus* osteomyelitis/discitis: focal paravertebral soft tissue abnormality, partial disc involvement, back pain for 10 or more weeks, and current antibiotic use for 1 week are predictive of fungal osteomyelitis/discitis (22).

Biopsy and histopathologic assessments are critical in the diagnosis of fungal infections. In this case, the previous candidemia was a determinant to start empirical antifungal treatment. There are no treatment guidelines or evidence-based recommendations for candida spondylodiscitis. The optimal medication and treatment duration are unknown. The Infectious Diseases Society of America and The European Society for Clinical Microbiology and Infectious Disease have established guidelines to prevent and treat nosocomial invasive candidal infection and osteomyelitis. They recommend a long-lasting treatment with 400 mg of fluconazole (6 mg/kg) daily for 6–12 months or echinocandin for at least 2 weeks followed by fluconazole. Another alternative is the lipid formulation amphotericin B at 3–5 mg/kg daily for at least 2 weeks, followed by fluconazole at 400 mg (6 mg/kg) daily for 6–12 months, but it has a higher risk of nephrotoxic damage. There are no guidelines about imaging or blood sampling follow-up.

Surgical criteria are well-described for the management of spinal infections. Neurological deficit or spinal instability is the foremost indication for this treatment, although there are other reasons. Among them are sepsis or abscess formation, inability to identify the microorganism responsible, inability to eradicate the infection by medical management, and intractable pain localized to the involved area of the spine (6, 23–27).

In our case, surgery was decided due to the infection's progression and the inability to control it with medical treatment. This caused worsening of the pain, increased thoracic kyphosis, and radiological compression due to an anterior epidural abscess. The patient remained neurologically intact.

There is no general agreement in the current literature about the approach and reconstruction method. Thorough debridement, maintenance of spinal stability, and anterior procedures are generally considered for the treatment of these lesions, while other issues remain controversial, such as the choice of the structural graft, the use of instrumentation in the infected spine, and same-day anterior-posterior vs. staged procedures (6, 23–25, 28). Surgical management of spinal infections can be challenging. Vertebral bodies and disks are the main elements affected, with posterior elements being frequently involved. This makes posterior approaches less suitable, and anterior approaches firmly recommended (2, 26, 27). Anterior approaches provide better access to the anterior spinal column, posterior band elements can be preserved with further instability prevented, and there is less risk for neural elements. In contrast, anterior approaches may not be familiar to the spine surgeon, and an access surgeon may be required.

In our case, due to the vertebral body impairment, an anterior approach was used. A thoracic surgeon did the thoracotomy, while a spine surgeon carried out the corpectomy and reconstruction. The right approach was chosen based on the site of infection and previous radiological images. On the left side, the aorta was lateralized to the left of the vertebral bodies, hindering access to the spine. Moreover, an infectious prevertebral abscess surrounded it, and a possible intraoperative rupture due to the weakened arterial wall concerned us. The liver was carefully retracted downward without impeding the right-side approach.

An expandable titanium cage with a lateral fixation plate was the reconstruction method chosen after the corpectomy. Nonetheless, large autografts (iliac crest, ribs, and fibula) have been classically considered as the gold standard technique for this purpose due to their good fusion rate and healing mechanisms. Recently, the additional morbidity and the appearance of new materials have led to displacement of the traditional methods (2, 23, 24, 29, 30). On the other hand, allografts and synthetic materials are considered to delay bone fusion and increase sequestration rates (23, 24, 30). No clinical trial has elucidated the best option, so the use of one or another ultimately depends on the surgeon's preference.

Similarly, instrumentation has traditionally been considered a hindrance for the antibiotics and healing mechanisms. Nevertheless, studies have proven otherwise, with successful healing rates after instrumentation (3, 24, 27, 31, 32). Moreover, instrumentation is advocated when more than one level is reconstructed or when posterior elements are damaged (23). As in our case, anterior column reconstruction should be enough if the posterior column is intact and the deformity is mild (2). Lastly, same-day anterior and posterior procedures are now being done in opposition to staged procedures. The former have lower complication rates and are also preferred by the patients and their families (3, 27, 31, 32).

Finally, the overall prognosis in candida spondylodiscitis is good, with cure rates of ~85%. Prompt diagnosis and treatment are paramount for this (6, 7, 33). As in other types of spondylodiscitis, medical treatment is the key, although surgical treatment can have its role in certain circumstances. We contribute to the evidence by describing one more case of fungal spondylodiscitis management. No complications were reported from a surgical view. The approach, titanium implant, and anterior instrumentation used were well-tolerated, and good clinical results were obtained. This reaffirms the scientific evidence published about this subject. Nevertheless, caution should be taken, and long-standing follow-up should be done to avoid delayed cryptic infectious processes related to the hardware implants (24).

## Lessons

COVID-19 has struck the society in a way that no other current disease has ever done. It is expected to possibly result in new and unfamiliar diseases arising in the following months or years. We must be prepared to face them. Fortunately, despite the uniqueness of some, current treatments will allow us to overcome most of them.

## Data Availability Statement

The original contributions presented in the study are included in the article/supplementary material, further inquiries can be directed to the corresponding authors.

## Ethics Statement

Written informed consent was obtained from the individual(s) for the publication of any potentially identifiable images or data included in this article.

## Author Contributions

LM-G and OE-S were responsible for the writing of this case report. DG-P and GG-P contributed the bibliography. JD-F and IP corrected the manuscript. All authors contributed to this manuscript and approved the submitted version.

## Conflict of Interest

The authors declare that the research was conducted in the absence of any commercial or financial relationships that could be construed as a potential conflict of interest.

## Publisher's Note

All claims expressed in this article are solely those of the authors and do not necessarily represent those of their affiliated organizations, or those of the publisher, the editors and the reviewers. Any product that may be evaluated in this article, or claim that may be made by its manufacturer, is not guaranteed or endorsed by the publisher.
